# Emerging Transistor Technologies Capable of Terahertz Amplification: A Way to Re-Engineer Terahertz Radar Sensors

**DOI:** 10.3390/s19112454

**Published:** 2019-05-29

**Authors:** Mladen Božanić, Saurabh Sinha

**Affiliations:** 1 Department of Electrical and Electronic Engineering Science, University of Johannesburg, Auckland Park, Johannesburg 2006, South Africa; 2 Research and Internationalization, University of Johannesburg, Auckland Park, Johannesburg 2006, South Africa; ssinha@uj.ac.za or ssinha@ieee.org

**Keywords:** terahertz band, millimeter waves, InP, SiGe, BiCMOS, transistor modeling, HBT, HICUM, SoC, SoP

## Abstract

This paper reviews the state of emerging transistor technologies capable of terahertz amplification, as well as the state of transistor modeling as required in terahertz electronic circuit research. Commercial terahertz radar sensors of today are being built using bulky and expensive technologies such as Schottky diode detectors and lasers, as well as using some emerging detection methods. Meanwhile, a considerable amount of research effort has recently been invested in process development and modeling of transistor technologies capable of amplifying in the terahertz band. Indium phosphide (InP) transistors have been able to reach maximum oscillation frequency (*f_max_*) values of over 1 THz for around a decade already, while silicon-germanium bipolar complementary metal-oxide semiconductor (BiCMOS) compatible heterojunction bipolar transistors have only recently crossed the *f_max_* = 0.7 THz mark. While it seems that the InP technology could be the ultimate terahertz technology, according to the *f_max_* and related metrics, the BiCMOS technology has the added advantage of lower cost and supporting a wider set of integrated component types. BiCMOS can thus be seen as an enabling factor for re-engineering of complete terahertz radar systems, for the first time fabricated as miniaturized monolithic integrated circuits. Rapid commercial deployment of monolithic terahertz radar chips, furthermore, depends on the accuracy of transistor modeling at these frequencies. Considerations such as fabrication and modeling of passives and antennas, as well as packaging of complete systems, are closely related to the two main contributions of this paper and are also reviewed here. Finally, this paper probes active terahertz circuits that have already been reported and that have the potential to be deployed in a re-engineered terahertz radar sensor system and attempts to predict future directions in re-engineering of monolithic radar sensors.

## 1. Introduction

Terahertz waves (THz waves) or submillimeter waves are defined as waves with frequencies in the range between 300 GHz and 3 THz [1], lodged between millimeter waves (30 GHz to 300 GHz) and infrared radiation [2,3]. This part of the spectrum has often in the past been referred to as the “terahertz gap”, given that for a long time it escaped the interest of both electronics and photonics researchers. In certain contexts, the low-THz band is also defined as situated between 100 GHz and 1 THz [4]. The potential of the THz band is great. The THz band is an excellent part of the spectrum for spectroscopy, as different materials show different absorption spectra at THz frequencies [5,6,7]. When it comes to organics, many macromolecules such as protein and DNA have vibrational modes in this part of the spectrum [8]. Unlike X-rays, for example, THz photons are non-ionizing and hence are not hazardous to living tissues and DNA, and the amount of radiation exerted by THz imagers is several orders of magnitude lower [3,9].

In the past two decades, a sharp rise in interest in technologies and applications operating in the low-THz and THz bands has been evident. Emerging spectroscopy and sensing THz applications include imaging, remote sensing, security and safety screening, process monitoring, non-contact/non-destructive materials testing, biological, medical and pharmaceutical analysis, label-free probing of genetic material, indoor mapping and navigation, target detection, as well as many others [1,4,5,10,11,12,13,14,15]. Various technologies and combinations thereof can be deployed in applications such as space exploration, weather studies, military and automotive applications, medical diagnosis research and security. In the developing world, furthermore, remote microsensing may have sustainability advantages [16]. The THz band is also suitable for ultra-fast communication; however, at present telecommunication research is still predominantly focused on the millimeter-wave band (for example, with the world-wide deployment of 5 G cellular networks, the millimeter-wave band is only starting to be utilized at the time of writing this article [17]) and thus there is no immediate need for exploiting THz communication and research in this area is still in its infancy, at least for the purposes of this review article.

Radar sensing in the THz band is explored because it has numerous advantageous characteristics over sensing in the millimeter-wave band. While in automotive applications, the millimeter-wave radar is increasingly exploited (for example, for park distance control where radars operating at 77 GHz are used), smaller wavelengths of THz waves (in the range of 1 mm) allow for higher spatial resolution and smaller footprints [18]. As a result, THz radars could potentially be used on small drones and even wearables. However, as more pressure is put onto increasing safety in vehicles, the number of integrated automotive sensors and their features is ever increasing [19]. Radars operating in the millimeter-wave regime, in addition to the above-mentioned park distance control, can also be used for adaptive cruise control, blind spot and lane change assistance, among other applications, but as research is moving towards self-driving, this is not sufficient. The current approach to self-driving assumes that cameras and lidars can be deployed for terrain sensing. Low-THz sensing, alternatively, can provide fine resolution images even in adverse conditions, such as fog, dust and snow, while also providing sensing image depth and insight into objects’ motion parameters [4]. In military environments, in addition to navigation benefits also applicable to automotive applications (and extended into avionics), THz radars can be used for small and multiple target detection [3]. Small target detection is also becoming important in space technology, for example, where detection of space debris is becoming problematic, as the amount of space junk is increasing [20]. Traditional detection of space debris is attempted with microwave radar or optical telescopes, which have a much lower resolution. What is not intuitive is that the small target detection is also associated with gesture recognition, which has already been explored using millimeter-wave radar for some time [21], but wider bandwidths and better range and resolution can be achieved with THz radar [13]. By sensing the time delay, frequency and phase shift and the amplitude attenuation of the radar signal (Doppler signature), real-time target properties, including distance, velocity, size, shape and orientation, can be attained and subsequently processed and interpreted.

THz imaging is another extremely important application of THz waves. A particularly important area of research is security, for example for concealed weapon detection in locations such as airports and train terminals. The main benefit here arises once again from the wavelength of the electromagnetic waves: although radiation with a wavelength of 1 mm can easily penetrate materials of which clothes are made, it is reflected by explosives [12], weapons [22] and drugs [23]. In general, passive imagers must be able to provide 1 cm resolution at a target distance up to 10 m, which is best achieved at frequencies below 2 THz and even better, 1 THz. Most airports today (late 2010s) are equipped with scanners operating at frequencies close to 30 GHz and the next generation of scanners is projected to use frequencies of close to 90 GHz [24], where a good compromise between the achievable image resolution and hardware costs can be found [23]. Real-time imaging, however, requires active imagers, which are suggested to be deployed in the THz range, around 300 or even 600 GHz [11]. Other attractive imaging applications are, for example, non-destructive scanning of mail packages. In food research, foreign particles, such as glass and other contaminants, can be detected by THz waves. Other attractive applications of THz waves are medical applications, where THz waves can supplement or even replace X-ray and ultrasound techniques [3].

Lack of efficient sources and detectors has meant that THz sensing, other than spectroscopy, has been slow to develop [25]. The main reason for this is that there are no commercially available transistor-based blocks, such as power amplifiers and low-noise amplifiers (LNAs). THz emitters are thus predominantly built using Schottky diode multipliers and mixers used to up-convert the signal towards THz frequencies [11,25]. Without LNAs, on the receiver side, signals in this approach can only be detected using mixers directly connected to the radar antenna. Another approach includes using broadband THz pulses generated by femtosecond lasers [24], with lasers being able to reach and surpass 1.5 THz [25]. In this and related (light-based) approaches, the transmitter illuminates the object and the receiver collects the scattered energy. Thermal THz detectors have also been used for detecting THz radiation, and early thermal THz detectors were predominantly based on bolometers [22]. In recent years, metamaterials with strong radiation-absorbing characteristics have also been investigated [5]. All of the above approaches lack the ability to amplify the signals properly, which is where the contribution of electronics has long been expected [26].

**Figure 1:**
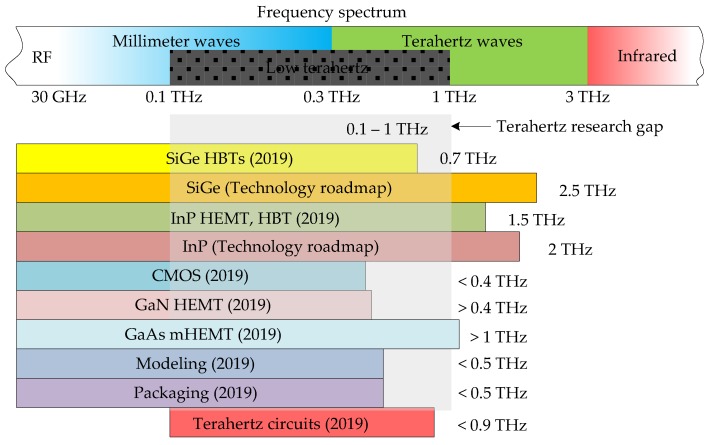
The THz research gap in relation to the present [29,31,41,42,43] and forecast [34,35] *f_max_* capability of transistor technologies, estimated current state of transistor modeling [44,45] and packaging [38] and reported achieved frequency operation of some THz circuits [1,42,46,47,48,49,50,51,52].

A fully electronic approach to THz detection has been researched for some time. However, the main bottleneck has traditionally been the lack of transistors capable of amplification in the THz gap. With inexpensive complementary metal-oxide semiconductor (CMOS) and bipolar CMOS (BiCMOS) technologies typically having transistors with transitional frequency (*f_T_*) and maximum oscillation frequency (*f_max_*) between 200 GHz and 300 GHz in the past [27], the only way to extend the operation of circuits into THz applications was deploying harmonic generation techniques [28]. Fortunately, the situation has recently started to change. Indium-phosphide (InP) transistors have been able to reach *f_max_* values of over 1 THz for around a decade already [29], while silicon-germanium (SiGe) BiCMOS heterojunction bipolar transistors (HBTs) also crossed the *f_max_* = 0.5 THz mark around the same time [30]. The *f_max_* = 0.7 THz mark was also crossed recently, as a result of the DOTSEVEN project funded by the European Commission [31]. In all cases, the increase in the frequency of operation was achieved by decreasing transistor feature sizes, an approach that can be taken repeatedly until the physical limits are reached [32,33]. Technology pioneers have developed roadmaps for both InP-based and SiGe-based technologies—describing the steps that should be taken to devise technologies with transistors that achieve operation close to the physical limits. In each case, it is predicted that *f_T_* can reach 1 THz and *f_max_* can reach or surpass 2 THz [34,35]. This means that if only transistors are considered, active circuits can already reach low-THz frequencies now without any harmonic generation, and they have excellent potential to extend their regions of operation further to the complete THz band in future. This is graphically illustrated in Figure 1, where the THz research gap, in this case defined to overlap with low-THz frequencies (0.1–1 THz), is shown in relation to the frequency spectrum and the *f_max_* capability of available and forecast technologies.

The second bottleneck in THz research related to active devices is the fact that despite transistor technologies becoming more readily available, for large-scale research to be feasible, these need to be supplemented by accurate and compact models [29,36]. Given that model development requires a combination of theoretical analysis, experimentation and circuit characterization only after the fabrication process has matured, models often lag behind the actual technology for a few generations. The third barrier is the microelectronic (or rather, nanoelectronic [37]) packaging, which also needs to catch up with technology developments so that THz circuits can be taken from controlled laboratory environments into the real world [38]. Even state-of-the art circuits perform poorly if placed into a non-optimal package. The current (estimated) state of device modeling and nanoelectronic packaging is also illustrated in Figure 1. Fourth, considerations such as fabrication (and modeling) of passives and antennas [14,39,40] also have to be brought in line with technology advances if these are not to hinder the re-engineering of THz sensors with THz-capable transistors. Finally, cost aspects related to active and passive device fabrication, as well THz-related legislation, need to be brought into the picture as well.

This paper, hence, undertakes to review the current state of transistor technologies capable of reaching THz amplification, as well as all related concepts that enable utilization of these technologies: active and passive device physics, modeling, packaging, and cost. The paper is organized as follows: In Section 2, emerging technologies capable of operating in the THz regime are described. Section 3 takes a step back and discusses the traditional and some alternative modern approaches to THz sensor implementation, at the same time highlighting where the active-device approach can introduce the cutting edge. In Section 4, other enabling factors for THz research, such as various stages of transistor modeling, are discussed. Section 5 and Section 6 summarize the role of passives, interconnects, antennas and packaging, and look at emerging approaches to fabricate these. Section 7 briefly touches on commercial aspects of THz research. Section 8 reviews recently reported THz sub-systems, some almost reaching 0.9 THz (Figure 1), which have the potential to be used in the implementation of future monolithic radar sensors. A discussion of the reviewed material as well as the anticipated forward direction of the research on re-engineering of radar sensors is given in Section 9. Finally, Section 10 concludes the paper.

## 2. Emerging Transistor Technologies Capable of Operating in the Terahertz Regine

In the context of active circuits, the capability of a technology to operate, reaching certain frequencies, is normally associated with two metrics: *f_T_* and *f_max_*. Although both metrics are transistor operating frequency parameters, each is a result of a different physics effects. Thus, the aim of technology researchers is to increase both parameters simultaneously. To investigate the main origin of *f_T_* in bipolar transistors, one needs to recall the well-known *f_T_* relation [53], defined as the frequency at which the transistor current gain becomes unity:

(1) $$f_{T}=\frac{g_{m}}{2\pi \left(C_{\pi }+C_{\mu }\right)}\;,$$

where *g_m_* is the small-signal transconductance of the transistor and *C_μ_* and *C_π_* are transistor parameters, which will not be discussed here but should be known to the reader. The transit frequency is mostly influenced by the vertical profile of the transistor [54]. It can be assumed that the sum of *C_μ_* + *C_π_* is dominated by the base charging capacitance *C_b_* = *τ_B_g_m_*, where *τ_B_* is the boron-doped base transit time in the forward direction, resulting in a simplified approximate relation

(2) $$f_{T}=\frac{1}{2\pi \tau_{B}}\;.$$

This means that the transitional frequency should increase with the decrease in the base transit time, which is, in turn, decreased by reducing the base width. Often, the total forward gate delay is used as another technology metric, which encompasses all transistor switching delays, including the base charging time [55].

The maximum oscillation frequency, or the frequency at which the power gain is equal to unity (power gain cut-off frequency), is not only dependent on *f_T_*, but also on the physical resistance in the transistor base, *r_b_* [56]:

(3) $$f_{max}=\sqrt{\frac{f_{T}}{8\pi C_{\mu }r_{b}}\;}.$$

Thus, to increase *f_max_*, bipolar technology researchers are also looking to decrease the resistance in the base, which is achieved by scaling of transistor lateral features. Naturally, there is a fundamental limit associated with vertical and lateral scaling, and new and innovative techniques are required to get ever closer to the scaling limit, as will be seen later in this section.

If the *f_T_* parameter is considered on its own, its value is more applicable to analog and mixed mode circuits, which need to have large current gains, and thus the analog circuits are normally operated at frequencies several times lower than *f_T_*. This is particularly true for wideband amplifiers, whereas narrowband amplifiers can more easily be designed at frequencies close to *f_T_*. For millimeter-wave and THz circuits, the situation changes, as these circuits are reliant on power gain rather than current gain. This is true for both power amplifiers and LNAs, as well as oscillators. Experience has shown that *f_max_*, which is generally higher than *f_T_*, should be between 50% and 100% higher than the design frequency to achieve practical THz circuits [26]. This means that THz circuits can be operated above *f_T_*. Since both *f_T_* and *f_max_* are results of technology designs, the ratio of *f_max_*/*f_T_* is different for each technology. A technology is considered balanced when *f_max_* is about 1.5 times larger than *f_T_* [33]. The maximum oscillation frequency is usually worked out by extrapolation and confirmed by practical amplifier circuits, some of which will be discussed in Section 8 of this paper. To operate circuits close to or even above *f_max_*, harmonic generation has to be used, but in this review, it is argued that this may not be necessary in the near future, owing to the good properties of emerging technologies, as will be seen shortly.

Radio-frequency (RF) amplification was initially achieved with CMOS and other field-effect transistors (FETs), because of their initial superiority over pure bipolar transistors [57,58]. After a bipolar transistor with a wide-gap emitter with a pn-junction built from differing (hetero-) materials (an HBT) was introduced, HBT-based amplification gained popularity. HBTs, as opposed to gated structures, are able to retain higher gains and higher current densities with a frequency increase. BiCMOS, a technology with both bipolar and CMOS transistors available, was also subsequently introduced, but BiCMOS technologies with *f_T_* and *f_max_*, which truly simultaneously exceed 300 GHz and 400 GHz respectively, could not be realized until the end of the first decade of the 21st century [48]. For a number of years now, the best reported metrics have come from integrated circuits (ICs) built with HBTs, high-electron mobility transistors (HEMTs) and metamorphic HEMTs (mHEMTs) [59]. HEMTs, like HBTs, use heterojunctions, but the overall transistor structure corresponds to that of the FET. Some of the fastest HEMT transistors are built in InP technology, with *f_max_* often exceeding 1 THz. The fastest HBTs have also been built in the same material base [29], both supported by aggressive technology scaling and favorable material properties. InP is an example of so-called compound III-V materials (where the roman numerals III and V refer to the old numbering of the periodic system groups); however, active research into a compound semiconductor from group IV, viz. SiGe, has yielded only a slightly lower *f_max_* figure of 0.72 THz (when considered that the frequency spectrum has logarithmic properties). Incentive-driven research into SiGe BiCMOS technologies was justified because of two main BiCMOS advantages — the ability of FETs and bipolar transistors to co-exist in the same substrate, allowing analog, digital and RF circuits to be fabricated on the same die, and the lower cost of (Bi)CMOS.

The remainder of this section discusses the current status and the short-term predictions for several main technology competitors, starting with InP HEMTs and HBTs and SiGe BiCMOS. The performance of a technology is predominantly the consequence of the material used and its properties. The most important properties of some of the materials that will be used in the remainder of this section are listed in Table 1.

### 2.1. InP HEMTs and HBTs

**Figure 2:**
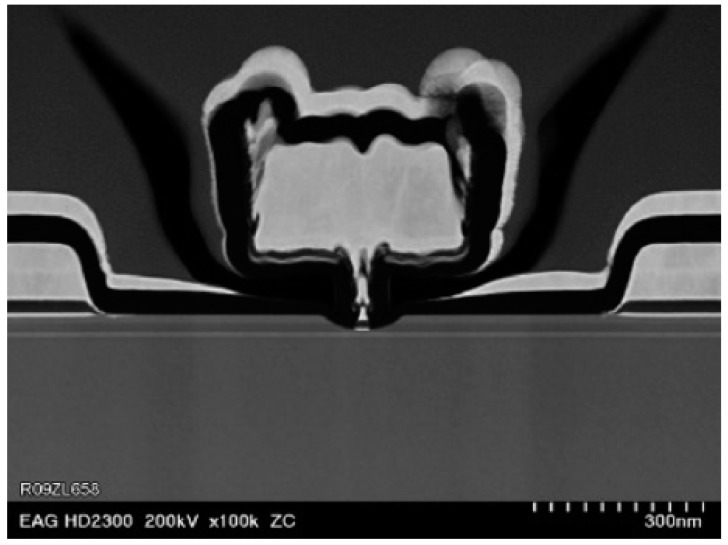
An electron microscope image of the 30-nm Indium-phosphide (InP) high-electron mobility transistors (HEMT) gate (reproduced with permission from [26]).

The main reason for the high performance of InP-based technologies is the combination of the wide gap of InP (1.34 eV) with the high electron drift velocity (more than 2 × 10^7^ cm/s) [62]. The key performance parameters of HEMTs include the gate length, the gate-source capacitance and the transconductance (which have a direct correspondence to HBT parameters in Equations (1) and (3)), as well as the FET-related parameter of the saturation velocity [2,59]. The first transistor with an *f_max_* of more than 1 THz was an InP HEMT transistor with a gate length of 35 nm, demonstrated in 2007. This technology also had an *f_T_* value of 400 GHz [26,51]. The *f_max_* value was confirmed by a practical demonstration of amplifiers operating at frequencies up to 850 GHz. The subsequent technology node was 30 nm, with *f_max_* = 1.3 THz and *f_T_* = 0.5 THz. In 2013, a 25 nm node was reported, with *f_max_* = 1.5 THz and *f_T_* = 0.61 THz, with amplification verified at 1.03 THz. To the best of the authors’ knowledge, this technology remains the technology with the highest reported *f_max_* to date. An electron microscope image of the 1.3 THz InP HEMT and its gate is reproduced in Figure 2. According to Deal et al. [26], high-frequency operation of this transistor was ensured by scaling of the size of the gate to 30 nm, resulting in reduction of the gate capacitance. The gate pattern was defined using electron beam lithography. The devices were fully passivated to a thickness of 200 Angstroms with plasma-enhanced chemical vapor deposition.

In addition to aggressive feature scaling (for low gate resistance), the high-performance HEMT was developed with epitaxial material enhancement for improved electron transport properties and process improvement. The composite channel was built with indium-arsenide (InAs) cladded between two indium-gallium-arsenide (InGaAs) layers, all lattice-matched. Various techniques were used to decrease the resistance of ohmic contacts and parasitic values such as source resistance. To achieve the precise alignment of the gate required for such small features, 100 kV e-beam lithography was used. This process includes high and low resistance thin-film resistors, metal-insulating-metal (MIM) capacitors and two metal layers for making connections. Even faster transistors should be possible if the indium composition inside the channel is increased, but the material becomes strained, and only thin layers are possible without dislocations. Such transistors are called pseudomorphic HEMTs [59].

Alternative InP transistor technologies are those incorporating double-heterojunction bipolar transistors (DHBTs). DHBTs also offer a wideband collector, allowing for a higher breakdown voltage at a given *f_T_*, which increases the drive current capability [29,49,55]. InP HBTs also have good transconductance and can be densely wired, but the key challenge is establishing stable low-resistance ohmic contacts. In HBTs, the transistor dimension refers to the emitter junction width. In 2016, 130 nm HBT technology was demonstrated in [55], showing *f_T_* = 0.52 THz and *f_max_* = 1.15 THz, and a common-emitter breakdown voltage of 3.5 V. The HBT was grown on a 100-nm InP substrate using molecular-beam epitaxy. The emitter is composed of InGaAs/InAs to minimize the contact resistance. The base is composed of carbon-doped InGaAs, while InGaAs/InAl/As is used in the base collector and base-emitter junctions. Benzocyclobutene (BCB), a dielectric, is used as a final passivation layer. The reported gate delay of this transistor is about 0.22 ps. The authors subsequently demonstrated amplification above 600 GHz. Alternative material composition can be sought from the InP/GaAsSb system [36]. GaAsSb has bandgap energy of 0.72 eV and its conduction band edge lies above that of InP, allowing for abrupt emitter-base and base-collector junctions to be implemented, which simplifies the DHBT epitaxial layer structure. This approach resulted in the state-of-the-art performance of *f_max_* = 1.2 THz and *f_T_* = 0.5 THz [64].

In the detailed technology roadmap published in 2008 [34], it was estimated that the 128 nm InP technology node would support HBTs with *f_max_* of 1.3 THz. This has been proven to correspond to the 130 nm technology node discussed above. In the same paper, it was extrapolated that the 64 nm node would be able to reach *f_max_* = 2 THz, and considering that the roadmap predictions held up to 128 nm, it is probably safe to assume that the trend is going to continue and that transistors with *f_max_* = 2 THz will become available in the near future. In fact, it was recently confirmed that no fundamental barriers exist that would prevent HBT dimensions from reaching 32 nm, which would open up the possibility for fabricating transistors with *f_max_* even higher than 2 THz and possibly *f_T_* higher than 1 THz [29].

**Figure 3:**
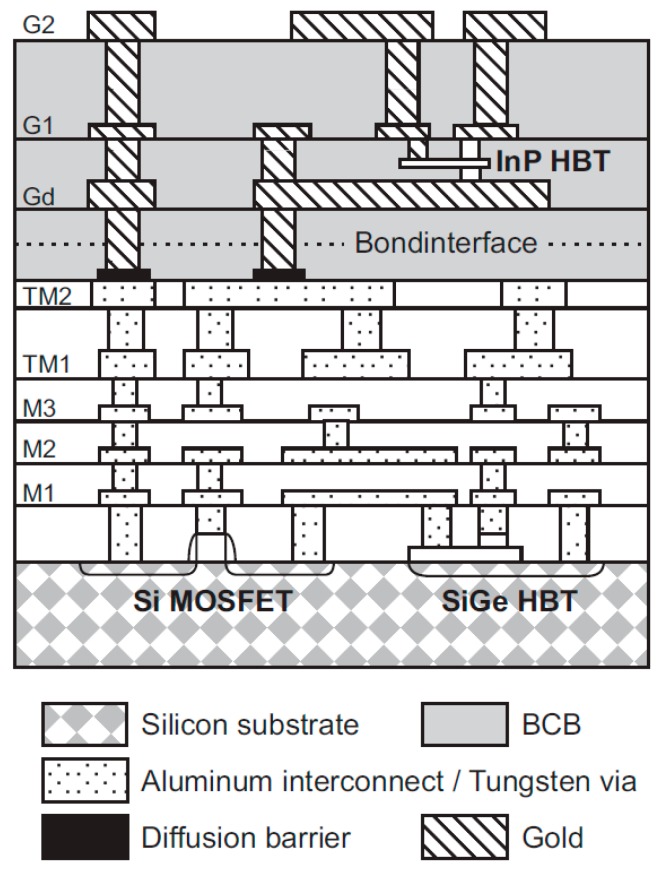
The final cross-section of the combined InP double-heterojunction bipolar transistors (DHBT) and silicon-germanium (SiGe) bipolar complementary metal-oxide semiconductor (BiCMOS) technology (reproduced with permission from [62]).

**Figure 4:**
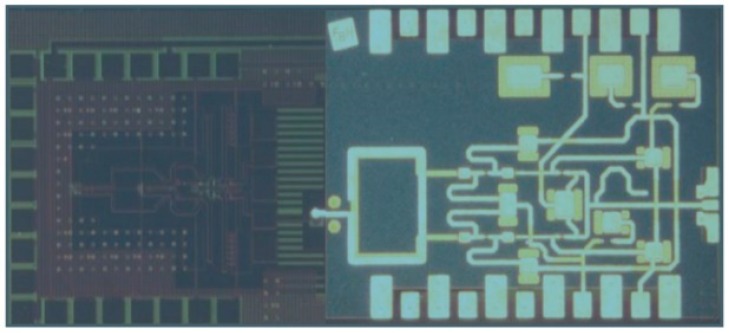
A microphotograph of the single chip, designed for the 328 GHz operation, showing a SiGe voltage-controlled oscillator (VCO ) on the left and the InP balun, buffer and a quadrupler on the right (reproduced with permission from [62]).

The biggest drawback of any III-V technology, including InP, has always been the limited variety of circuits that can be fabricated [62]. Pure HBT technologies, such as InP DHBT, would typically just be used to fabricate millimeter-wave or THz circuits, while the remaining circuits (e.g., digital circuits and the analog front end) would be fabricated in CMOS. This approach is typically costly, because multiple process technologies, packages and supporting boards would be used; furthermore, such a system would often experience high losses in signals transitioning different dies. In recent years, it was proposed that InP HBT technology could be integrated with SiGe BiCMOS technology (described in the following section), and such hybrid technology would be able to offer the benefits of both technologies. One of the possible methods is that presented by Weimann et al. [62], where processing is done in two fabrication facilities (fabs): a III-V fab and an SiGe fab. First, the BiCMOS wafer is fabricated in the SiGe fab. Then the processing continues in the III-V fab where another wafer, with InP DHBTs, some additional layers, including gold interconnects, low-loss dielectrics and thin-film resistors, is fabricated. The two wafers are then aligned and wafer-bonded together, face-to-face, using BCB as the adhesive, forming the bond interface, in the III-V fab. Prior to bonding, owing to differing wafer sizes, the larger 8” BiCMOS wafer has to be cut into three 3” wafers to match the size of the InP wafers. After bonding, the InP substrate is completely removed, leaving only InP semiconductor islands, thus resulting in a combined SiGe/InP BiCMOS technology with eight metal layers, Si MOSFETs, SiGe HBTs and InP HBTs, as shown in the cross-section in Figure 3. Note that in this method, deep tungsten vias, similar to through-silicon vias (TSVs), typically associated with packaged solutions, which will be discussed later in this article, are necessary to connect aluminum metal layers from the SiGe technology with gold metal layers from the InP technology, also shown in Figure 3. A microphotograph of the 328-GHz IC test vehicle designed by Weimann et al. is reproduced in Figure 4. The figure shows a SiGe voltage-controlled oscillator (VCO) on the left and the InP balun, buffer and a quadrupler on the right, illustrating how different circuitry can be distributed to take advantage of both technologies simultaneously (different substrates also appear in different colors in the figure). The approach by Weimann et al. indeed constitutes an excellent proof of concept, despite the fact that the *f_T_* and *f_max_* values of the InP technology are both lower than 400 GHz. However, as the smaller-feature InP processes mature, it should be possible to reproduce the approach for truly THz technologies.

### 2.2. SiGe BiCMOS and HBTs

Although it appears that InP technologies are more capable of reaching into the THz band than SiGe BiCMOS, it was seen in the previous subsection that the complexity of circuits that can be integrated is much lower. This is one of the main reasons for recent incentivized research efforts to produce BiCMOS technologies capable of reaching THz operation. Another reason is, naturally, the cost of BiCMOS fabrication, which has traditionally been the least costly technology when it comes to fabrication, alongside pure CMOS processes.

SiGe BiCMOS technology became capable of RF applications in the early 1990s, when 200 nm HBTs with *f_max_* = 100 GHz were fabricated [48]. Reaching that particular milestone was sufficient for applications such as cellular technology, running at 900 MHz, and wireless local area networks, running at 2.4 GHz, which required low-cost and small form factors, but not extremely fast transistors. The subsequent telecommunication boom in the early 2000s and the observed potential to explore the bottom of the millimeter-wave band triggered research of BiCMOS technologies that could reach 200 GHz and beyond. The most important commercial success of SiGe technologies at frequencies extending beyond 30 GHz was the commercialization of automotive radar operating at 77 GHz. In the 2010s technologies with *f_T_* and *f_max_* exceeding 300 and 400 GHz respectively were developed. The performance of RF CMOS technology was being improved (by virtue of aggressive scaling beyond 100 nm) in parallel to that of BiCMOS, but BiCMOS had the advantage of increased circuit complexity, since bipolar devices could appear with RF CMOS devices in the same circuit. HBTs in BiCMOS also provided for higher transconductance values and consequently higher output power levels than RF MOSFETs at the same drive levels and frequency. However, none of these technologies (CMOS or BiCMOS) was capable of reaching into the THz part of the spectrum. At that point it was realized that feature scaling alone was not sufficient for further improvement in the existing technologies, and a different approach needed to be taken [30,32,33]. The solution was to bring together the major European semiconductor industry key players, supported by the efforts of academic researchers, in a highly funded project that would investigate the steps needed to be taken to improve the *f_T_* and *f_max_* figures incrementally, until a technology with *f_max_* > 0.5 THz was found, while staying ahead of non-European competition. This project was named the DOTFIVE project (for 0.5 THz); it also had the secondary objective of reaching a gate delay as low as 2.5 ps.

**Figure 5:**
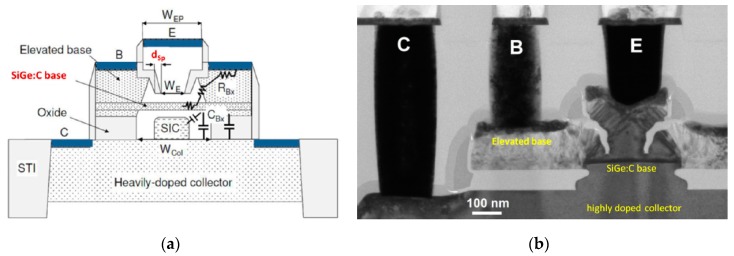
(**a**) Diagram and (**b**) electron microscope photo of the 0.5 THz heterojunction bipolar transistors (HBT) fabricated by IHP (reproduced with permission from [48]).

The DOTFIVE project ran in several stages, with initial incremental improvements on the existing self-aligned epitaxial base transistors in a SiGe technology from STMicroelectronics (*f_T_*/*f_max_* = 230/290 GHz). In the first stage, the aim was to shrink the sizes of the base, emitter and collector, reaching the technology that had HBTs with *f_T_* = 260 GHz and *f_max_* = 350 GHz [30]. In the second phase, silicon-germanium-carbon (SiGeC) HBTs were fabricated featuring *f_max_* > 400 GHz and reduced emitter and collector widths of 110 nm and 280 nm, respectively. Carbon co-doping was introduced to largely suppress boron diffusion, which in turn allowed a lower base width to be achieved [65]. However, any further improvements required conflicting parameter optimization, and even further base transit time reduction, as well as the reduction of the base/collector depletion layer transit time. This could only come from device architectures with inherently lower device parasitics. The subsequent approach to the 0.5 THz transistor included device profile optimization, various collector isolation schemes to decrease collector capacitances, and combining the self-alignment of emitter-base junction and base connection. The combination of approaches resulted in two HBT technology options from IHP: A 155-nm technology, with *f_T_* = 310 GHz, *f_max_* = 480 GHz and gate delay of 1.9 ps and a 120-nm technology, with a slightly lower *f_T_* (300 GHz) and slightly higher gate delay (2 ps), but with *f_max_* reaching the original predicted value of 500 GHz [66,67]. The integration of the 0.5 THz HBT into a BiCMOS technology was subsequently achieved. A diagram and electron microscope photo of the cross-section of the 0.5 Hz SiGe HBT are shown in Figure 5, taken from [48]. Figure 5a shows the primary technology features, which include the elevated SiGeC base (B), the heavily doped collector (C), selectively-implanted-collector (SIC) formation and the surrounding oxide, the narrow emitter (E) and the shallow trench isolation (STI). The widths (*W*) of various regions are also shown in this figure, together with capacitive (*C*) and resistive (*R*) parasitic quantities that remain despite the device scaling. In Figure 5b, the reference 100 nm scale can be used to get an idea of the size of the transistor features mentioned.

The DOTFIVE project was followed by the DOTSEVEN project [67], which had the main objective of extending the *f_max_* beyond 0.7 (.7) THz. Efforts to continue HBT scaling were justified in a two-part paper by Schröter et al. [32,33] published during the time of the DOTFIVE project, which assessed ways in which transistors could be vertically and laterally scaled further, possibly even leading to technologies with *f_max_* = 1.5 THz, while retaining a collector-emitter breakdown voltage over 1 V. Finally, a transistor with *f_T_* = 505 GHz, *f_max_* = 720 GHz, breakdown voltage of 1.6 V and a gate delay of only 1.34 ps was reported in 2016 [31], achieving for the first time *f_T_* and *f_max_* that were simultaneously larger than 500 GHz in a SiGe technology [68]. This performance was achieved in two iterations. In the first iteration, the non-epitaxial growth of the SiGe base layer was used to form the HBT structure, instead of the traditional double-polysilicon self-aligned structure. This was supported by the boron-doped base link module, which allowed for *f_max_* to be improved to 550 GHz with *f_T_* = 330 GHz. To improve the *f_T_* and *f_max_* values to the state-of-the-art values above further, several additional steps were introduced, such as an in situ doped epitaxial external base, the elimination of the base link anneal, and an ms flash anneal. The 0.7 THz HBT process retained functional metal layers, MIM capacitors, metal-oxide semiconductor varactors and polysilicon resistors and although the challenge of integrating HBTs in advanced CMOS technology remains [54] unsolved, the two technologies are deemed compatible.

The 0.7 THz transistor fabrication was the culmination of the DOTFIVE and DOTSEVEN projects, and their successful outcome was heavily supported by extensive physics-based modeling and numerical as well as electrothermal simulation tools. The only marginally better SiGe HBT was reported by Charaborty et al. [69], having an *f_max_* of 798 GHz, but this result was achieved only at unpractical cryogenic temperatures. However, what this shows is first, that among other implications, the HBT performance can be increased by cooling, and second, that the performance limits of SiGe-based technologies have not been reached yet.

The latter of the two conclusions is further supported by the SiGe HBT technology performance scaling roadmap presented by DOTFIVE/DOTSEVEN researchers in 2016 [35], at the conclusion of the two projects. The roadmap was devised using the results of various 1D, 2D, 3D technology simulators combined with compact modeling, which included all known parasitic effects and effects such as self-heating. Realistically achievable technology scaling nodes were proposed, based on the information on the currently available fabrication hardware and predictions for its future scaling, as well as the predicted physical transistor limits. According to the prediction, *f_max_* between 2 THz and 2.5 THz could be realistically achieved with the transistor dimensions scaled to about 20% of the 0.5-THz-transistor dimensions published as a result of the DOTFIVE project. However, a number of challenges were identified for further technology scaling, of which some will be mentioned here. First, the vertical structure needs to continue to shrink, which is associated with the requirement to increase the doping in order to keep the resistivity sufficiently low. Then, the base layer will have to be grown with atomic-level precision, which requires further fabrication technology advancement. Novel epitaxy approaches will also have to be implemented to manage complex collector profiles, and co-doping by materials other than carbon would need to be suggested to suppress boron diffusion further. Further sheet resistance reductions would be needed as well to allow high *f_T_* and *f_max_* at the same time and the emitter resistance will need to be decreased further. Such tiny emitter (and transistor) structures are furthermore expected to suffer from electromigration effects, which will need to be alleviated. Alternative contact materials and metallization will be sought too in order to increase the achievable current density. Finally, it must be noted that the technology modeling will have to follow the technology scaling closely so that each new node iteration can be understood and evaluated properly.

### 2.3. Si CMOS

CMOS technology, traditionally used in digital and low-frequency analog circuits, has been quite capable of competing with the BiCMOS technology, at least up to millimeter-wave frequencies [70], and may be found suitable for THz applications in the near future. CMOS is favored owing to the low cost of fabrication and high level of achievable integration. According to Lee [71], the maximum *f_T_* of a CMOS technology can be approximated (to the first order) by the formula *f_T_* = 10 (THz·nm)/*L*, where *L* is the gate length in nanometer, meaning that the *f_T_* of a 5 nm technology could be 2 THz, if the length can be feasibly decreased to such low levels. Considering the challenges of transistor optimization listed in Section 2.2, reaching such frequencies even at such short lengths is unlikely. A better prediction is perhaps the one of Ellinger et al. [72], who extrapolated that *f_T_* of 500 GHz would be possible at a gate length of 10 nm. Certain process modifications from standard CMOS are, however, needed to achieve good performance. A possible path forward could be sought in the tri-gate (FinFET) structures introduced relatively recently as part of efforts to decrease short-channel effects in FETs [73,74,75].

The best-performing CMOS technologies (in terms of *f_T_* and *f_max_*) are typically based on the silicon-on-insulator (SOI) principle [41,76]. SOI circuits are composed of single-device islands, which are isolated from the substrate and from each other [77], which reduces transistor source/drain junction capacitances and substrate coupling of passives. Transistors are also less prone to short-channel effects and can perform better at lower voltage supplies. A 45-nm IBM process is the state-of-the-art example of the CMOS SOI process with *f_T_* and *f_max_* both above 250 GHz [41]. Initial results indicated that *f_T_* of almost 0.5 THz (485 GHz) would be possible [78], but the limitations of the body-connected SOI FETs meant that layout optimization resulted in halving the *f_T_* value. THz applications may benefit even more from high-resistance SOI (HR-SOI) technologies. HR-SOI technology differs from regular SOI technology in the sense that the substrate is modified to have resistivity of more than 1 kΩ·cm [79]. This is important in the design of passives and interconnects (both discussed in more detail in separate sections later). Transmission lines, such as coplanar waveguides (CPWs) are used in integrated designs at millimeter-wave frequencies and beyond instead, and if high resistivity of the substrate is used, transmission-line losses are decreased. This effect occurs owing to the decrease in line resistance per unit length due to high substrate resistivity.

Another suggestion to improve CMOS performance is to introduce strained silicon [72]. This is done, for example, by placing the active silicon layer on top of another material with a larger lattice constant, such as SiGe. This results in higher carrier mobility of the transistor channel. The performance of CMOS can also be improved by cooling, as was shown to be the case with BiCMOS [69], or by increasing the dielectric constant of the gate oxide (e.g., replacing silicon-dioxide by high-*k* oxide). Silicon-nanowire FETs could also be used as an alternative to regular FETs, but many challenges in their practical high-frequency implementations remain unsolved, although these transistors can be used for passive detection, as will be seen later.

### 2.4. Gallium-arsenide (GaAs) mHEMTs

GaAs HEMTs generally suffer from low integration capabilities, but GaAs substrate is more robust and more affordable than InP substrate. Good crystal quality and good overall technology performance can be achieved if metamorphic transitional layers are used to connect the substrate to active device layers built out of InP, InGaAs or InAlAs [59]. Resulting mHEMTs yield performance similar to that of regular HEMTs and *f_T_* values above 500 GHz as well as *f_max_* values above 1 THz are readily achievable [42]. In mHEMTs, each of the base, collector and emitter is composed of a different alloy, unlike regular HEMTs. In addition to cost savings, GaAs-based technologies also have good noise performance [50].

### 2.5. GaN HEMTs

GaN technology has the advantage of supporting HEMTs that are much smaller in size than devices fabricated in other technologies for the same output power. The wide bandgap (3.32 eV) of GaN also enables transistors to operate at high temperatures, while yielding good noise performance. This makes GaN technology quite suitable for both high-power applications (power amplifiers), as well as low-noise applications (LNAs), albeit at higher costs than that of other technologies [80]. This is particularly true when comparing it to SiGe, which can also yield moderately high output power and fairly low noise performance. However, in applications such as planetary exploration, having high output capability is more important than the cost itself [81]. In terms of frequency performance alone, an impressive *f_T_*/*f_max_* combination of 454/444 GHz was reported in [43], as a result of reducing fringing gate capacitances by optimizing the epitaxial layer thickness and carrier distribution. A further increase in speed should be achievable by decreasing mesa-edge capacitances that form during etching as a result of mesa isolation and present a large portion of the total gate capacitance [82]. The possibility of growing GaN double heterostructure high-electron mobility transistors on silicon wafers was also explored for the purpose of increased integration complexity [83].

## 3. Traditional and Current Approaches to Terahertz Radar and Direct Terahertz Sensing

A large number of THz radar and sensing approaches that do not use solid-state amplification have been reported to date, with new techniques continually emerging. Some of these approaches are reviewed in this section.

### 3.1. Laser and Schottky-based Detectors

**Figure 6:**
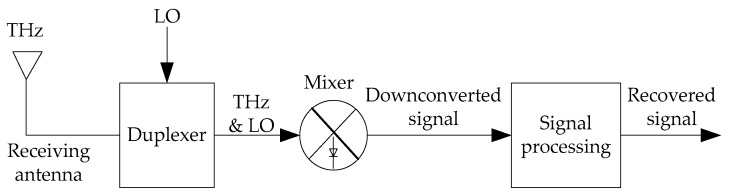
Illustration of the radiation detection deploying a Schottky diode mixer (adapted from [86,87]).

The earliest attempts at THz imaging date back almost 50 years. One of the first systems involved carbon-dioxide far-infrared laser on the transmitter side to generate THz radiation, and a bolometer on the receiver side to measure the amplitude of the scatter [84]. Over the years, however, the most popular approach to THz detection became the one involving two-terminal semiconductor diodes based on Schottky barriers in GaAs and InGaAs semiconductor technologies [85]. Originally, diode mixers were used for frequency multiplication and heterodyne detection, but these can also be used as transmit elements in radars, to up-convert signals to the THz frequency of interest. As far as detection goes, Schottky diode mixers now dominate in all commercial detectors, and have long been used at frequencies as high as 1.5 THz [84]. A simplified block diagram of the diode-based detector is shown in Figure 6, illustrating the path the detected signal traverses [86,87]. Both local oscillator (LO) and THz radiation are applied to the diode mixer simultaneously, and the result is a down-converted signal, which can be filtered and amplified with the RF electronics operating around the frequency of LO.

Systems with Schottky emitters and detectors are regarded as all-electronic systems. On the other hand, photonics systems (systems based on lasers [63]) have their advantages, but still typically require passive receivers, as was the case at the very beginning of THz imaging. Typically, laser imagers can deploy the pulsed wave or continuous wave approach [88]; the pulse-wave approach has been used for many more years and is the much more widely adopted one of the two. The continuous-wave approach is the current focus of research, returning a higher level of image depth information, but requires active systems. Synthetic-aperture imaging and synthetic-aperture radar are two approaches to active imaging [11,85,89].

The simpler pulsed imaging is similar to ultrasonic imaging and involves emitting a short pulse of laser radiation at an object of imaging, and recording of the time delay of reflections to form an image [84]. An all-laser approach has, however, been explored, removing the need for an external detector [90]. It involves a laser system in which the laser radiation reflected from the target is re-injected into the laser cavity, resulting in interference between the intra-cavity field and the re-injected signal, which can be monitored. Such an approach has been termed laser-feedback interferometry.

What is common to all the above imaging approaches, in addition to the lack of solid-state amplification at the frequency of radiation, is that the systems tend to be bulky and expensive, often involving multiple technologies, techniques and discrete components combined to build the required sensing apparatus. Sensing using active components increasingly capable of THz operation, as described in Section 2, on the other hand, has the potential to overcome all of the aforementioned challenges by replacing traditional systems with custom-designed inexpensive monolithic solutions. Exceptions to the rule are modern detection approaches, discussed next.

### 3.2. Modern Detectors and Sources

Modern detection approaches involve metamaterials [5], microelectromechanical systems (MEMS) detectors [22], modulators based on graphene and other 2D semiconductors, as well as passive detection using traditional transistors (HBTs and HEMTs), with many new approaches demonstrated post-2010.

For the nanoelectronic community, the case of the graphene FET (GFET) is particularly interesting. Graphene has superior carrier mobility and belongs to a group of 2D semiconductors, crystalline materials having only a single layer of atoms. GFET is a gated structure similar to regular FET, with a graphene channel laid on top of a silicon or alumina substrate. Graphene is intrinsically a zero-bandgap semiconductor, and as such a GFET cannot be used as a traditional transistor [91,92]. Large-area GFETs can, however, be used as wideband THz modulators, as they are highly sensitive to free carrier concentration, while the gate allows for effective tuning. For example, plasmon-assisted resonant detection of THz radiation by antenna-coupled graphene transistors that act as both plasmonic Fabry-Perot cavities and rectifying elements was demonstrated by Bandurin et al. [93] for the low-power radiation at 2 THz. It was also shown that graphene-based ring resonators are suitable for sensing. In their paper, Zangeneh-Nejad and Safian demonstrate label-free probing using a silicon-nitride (Si_3_N_4_) dielectric ring resonator vertically coupled to a graphene-strip ring resonator on top in [8], as well as a slightly different resonator where Si_3_N_4_ is coupled to a thin layer of silver with a hybrid graphene-MoS_2_ layer on top of this structure in [94]. Finally, it should be noted that graphene also has tunable optical properties, which can be used to build reconfigurable devices [95]. This can be an advantage to the bandwidth limitation of THz transistors imposed by the Kramers-Kronig relation [96].

A new class of 2D elemental materials, such as silicene, germanene and phosphorene, may also be found suitable for THz detection [97]. Among these, black phosphorus, which is the most stable allotrope of the phosphorus element in standard conditions, has a layered graphite-like structure. Photodetection with black phosphorus was demonstrated in a wide band around 0.3 THz recently by Viti et al. [97]. The 1D InAs nanowire FETs are also suitable for detection, as demonstrated by Vitello et al. [98], once more around 0.3 THz.

Active semiconductor devices can also be used for direct THz radiation detection, even if the radiation is above *f_T_* and *f_max_*. Both HBTs and HEMTs on InP substrates, for example, can still rectify THz voltage and current up to about 3 THz despite having no available gain at these frequencies [99]. THz detection using traditional CMOS has also been demonstrated, utilizing the phenomenon of the Dyakonov-Shur plasma wave theory [100]. In a paper by Schuster et al. [101], 130-nm Si MOSFETs were used for detection in the wide frequency range spanning 0.27 to 1.05 THz. In another contribution by Ikamas et al. [102], CMOS detectors are used for the detection of laser radiation at 3.1 THz. Detectors based on GaN HEMTs have also recently been demonstrated. In a paper by Čibiraitė et al. [103], broadband detection between 0.2 and 1.2 THz was demonstrated, with the most remarkable performance being achieved at 0.5 THz. Ikamas et al., Čibiraitė et al. and Vitello et al. all argue that the use of a bow-tie type antenna increases detection efficiency.

Antennas are, evidently, another way to boost the efficiency of detectors and emitters. In [104], it was shown that the already mentioned hybrid graphene-MoS_2_ strip can be used as an antenna if deposited on GaAs substrate. Such a structure shows high values of matching efficiency and radiation efficiency.

With the conventional photo-emitters exhibiting low output powers, in the order of microwatts only, considerable effort has been made to increase their efficiency as well. Ironside et al. [105] show that by forming a metal-semiconductor-metal cavity through layering of an ultrafast semiconductor material between subwavelength metal-dielectric gratings, a photomixer can be enhanced to generate powers in the milliwatt range for frequencies between 1.2 and 1.8 THz. The photomixer, in this design, is connected to an E-patched antenna to boost efficiency.

## 4. Modeling for Re-Engineered Terahertz Research

The availability of adequate transistor technologies is only the first of several enabling factors leading to re-engineering of THz systems. When a new technology emerges, the first, usually extrapolated, results are usually swiftly reported to the scientific community. A second step is the fabrication of circuits that serve as the proof of concept that the claims of the technology are valid, and the initial fabricated systems are designed and analyzed by the same research group that developed the technology. However, before the technology can be widely adopted by other researchers and subsequently commercialized, the physics of the technology needs to be understood, followed by the development of technology simulation models that open up the possibility of first-time prototyping success. Therefore, physics and modeling of THz-capable technologies are understood to be the second enabling factor for re-engineering THz systems and are subsequently discussed.

### 4.1. Physics-Driven Small-Signal Modeling of Transistors

It is common knowledge that the research and design of electronic circuits, in general, are performed in several stages, where a first stage typically involves looking at and understanding the analytic model that is comprehensible to designers without consulting electronic design automation (EDA) [106] tools. Transistor behavior is normally described by small-signal models, which are generally applicable to most signals in analog circuits and some signals in RF circuits. Specialized circuitry, such as power amplifiers (operation at much lower frequency than *f_T_* is assumed here), requires transistors to operate in large-signal modes, and large-signal models are more applicable in such cases.

**Figure 7:**
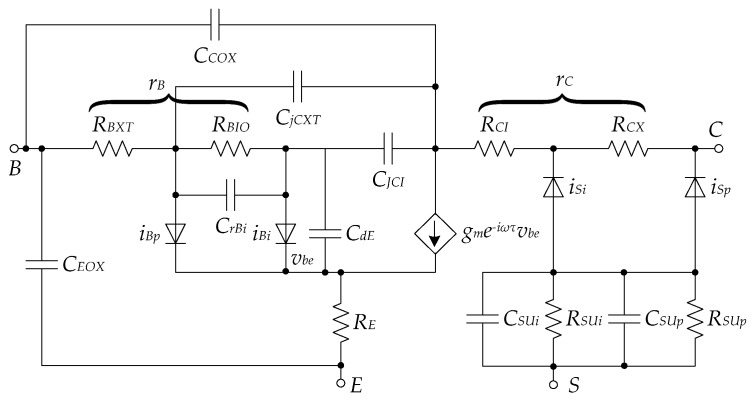
A small-signal model of the HBT applicable at millimeter-wave frequencies up to roughly 110 GHz (adapted from [107]).

For every transistor, irrespective of the technology node, a range of simple or complex small-signal models can be developed; however, simpler mathematical equations based on the device-physics alone struggle to describe the transistor behavior accurately beyond a certain model-dependent frequency. The small-signal model of the HBT in Figure 7, for example, breaks down the transistor up to the level where each pn-junction that is formed is modeled in detail by current and periphery parameters (diodes *i_Bp_*, *i_Bi_*, *i_Si_* and *i_Sp_*) [107]. The model also includes internal and external bias-dependent capacitances between different terminals (*C_jCXT_*, *C_JCI_*, *C_rBi_*, *CdEi*, *C_SUi_*, *C_Sup_*, *C_COX_* and *C_EOX_*), resistances of the base (*r_B_*), collector (*r_C_*), emitter (*R_E_*) and substrate (*R_Sui_* and *R_SUp_*), as well as a voltage-dependent current source. This model is much more comprehensive than typical small-signal high-frequency models found in various textbooks (e.g., [53]), which usually include only one voltage-dependent current source and fewer resistances and capacitors. Despite the level of detail of the model in Figure 7, it is still expected that the high-frequency effects will compromise the validity of the model beyond 110 GHz. Beyond this frequency, designers rely on empirical models, such as ones based on, for example, large-signal *S*-parameter extraction or newer *X*-parameters extraction (a variation of *S*-parameters extraction relying on a more realistic large-signal excitation [108]), rather than expanding the small-signal model to take into account more complex effects. It is thus clear that for THz research, understanding of physics phenomena is merely a beginning, and that research depends heavily on more advanced models, which are unpractical for use by hand, but replace both physical and empirical small-signal models. The use of these models is only feasible in conjunction with EDA tools; that is, in simulation.

### 4.2. Simulation Models

Simulation models of bipolar transistors date back to around 50 years ago when the SPICE Gummel-Pool model (GPM) was reported [109]. Most commercial simulators at the turn of the century still supported GPM as the main bipolar simulation model [110], even though a more accurate vertical bipolar inter-company (VBIC) model had already been developed as the replacement industry standard model for several years [111]. At that time it was already well established that GPM does not correctly describe the transistor in the high-current region or at low voltages, which made it unsuitable for high-frequency (RF) applications [112]. The VBIC model does feature a number of improvements; however, its charge storage and transit time models are based on the VBIC equations, meaning that it still suffers from many of the same problems associated with the GPM. With the emergence of technologies specifically geared towards RF operation and beyond, a clear need for more accurate models that are able to follow rapid technology scaling has been identified.

**Figure 8:**
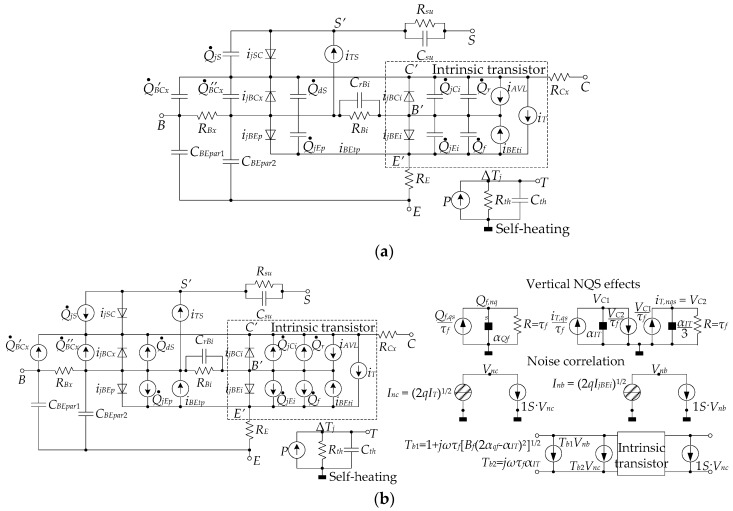
The evolution of high-current model (HICUM): (**a**) HICUM/L2 in 1999 (adapted from [115]) and (**b**) HICUM in 2015 (adapted from [44]).

At present, it is generally considered that the scalable high-current model (HICUM) [113] is able to follow the HBT (or bipolar in general) technology scaling with a lag of only a few technology node generations (to allow for model extraction for a wide range of transistor sizes and configurations to be performed). The main characteristics of this physics-based model is its ability to be expanded by either adding new model parameters [44] or updating the old parameters [114] as technologies evolve and new device physics effects are discovered by simulation, characterization or experimentation. Figure 8 shows two variations of HICUM level 2 (L2) developed around 15 years apart, in 1999 [115] and 2015 [44] respectively. The model from 1999 (Figure 8a) is already much more comprehensive than the small-signal model presented in Figure 7, and it was suitable for HBTs with the intention of operating the microwave range at the time of development. The number of diodes, capacitors, resistors and dependent sources was increased when compared to best analytic high-frequency models (Figure 7). Furthermore, the transistor was split into an intrinsic (internal) and an external part. The model included effects such as collector avalanche breakdown (*i_AVL_*), base-emitter tunneling (*i_BEt_*), self-heating (separate network as indicated in the figure), substrate coupling and the effects of the parasitic substrate transistor. The recent (2015) version of the model (Figure 8b) repeats the main and self-heating networks of the model from 1999, and also includes non-quasi-static (NQS) effects occurring at high frequencies by virtue of adding another separate network. The evolved model is applicable to transistors capable of millimeter-wave and, more recently, THz operation. This version also considers the effects of the correlated noise, added as a result of research reported in [116]. It is expected that the model will continue to evolve; for instance, shortly prior to the publication of this article, HICUM was extended to include the avalanche multiplication in SiGe HBTs at different transistor injection levels [117]. A detailed description of HICUM, including the definition of all symbols used in Figure 8, can be found in the HICUM/L2 manual by Schroter and Pawlak [113]. This manual is available as a direct download from the website of Dresden Technical University, where the model originated.

HICUM development was extensively revised during the course of the DOTFIVE and DOTSEVEN projects and parameters were extracted for each reported technology node [36]. HICUM/L2 is now available in all commercial simulators and is at present implemented in Verilog-A. Although it was originally developed with SiGe BiCMOS technology scaling in mind, model parameters can be extracted for any HBT technology, for example for any of the contending InP HBT technology nodes [36,118]. The methodology of parameter extraction is also detailed in the HICUM/L2 manual [113].

When it comes to modeling of HEMTs, the situation differs between specific technologies. Modeling of InP HEMTs, the fastest of all HEMTs, has not been extensively reported upon in literature, unlike, for example, GaN HEMTs used in high-power amplification [119,120]. Commercial simulators still use InP HEMT models that date back to the late 20th century, while the more recent models are based on extrapolation from modeling of GaAs or GaN HEMTs [121,122]. However, some attempts at non-linear large-signal modeling of InP HEMTs have recently been made [121], but it becomes increasingly difficult to separate device behavior from the electromagnetic environment [123]. Design in this technology is then typically performed using empirical models. The situation with CMOS FETs is, fortunately, in a better state, as a range of Berkeley short-channel IGFET models (e.g., BSIM4) has been kept up to date with technology scaling [124]. However, by noting again that CMOS is currently still lagging behind HBT and HEMT technologies in terms of achievable frequencies, it would appear that HBT technologies (including SiGe BiCMOS) remain the only contenders that can currently reach into the THz domain, with supporting simulation transistor models that are able to do the same.

### 4.3. Summary—Terahertz Technologies, Modeling and Cost

**Figure 9:**
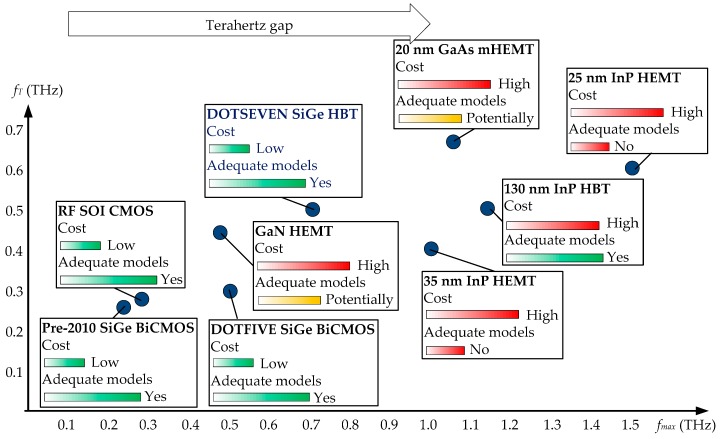
Comparison of emerging transistor technologies capable of THz applications with respect to performance metrics, extent of modeling and cost. Linear frequency axes are used for clarity of presentation. From left to right, technology metrics have been taken from these references: [26,29,31,41,42,43,48,55,66].

Figure 9 shows the performance metrics of reported THz technologies, together with their estimated fabrication costs and the extent of modeling of their active devices. This figure reassures one that SiGe BiCMOS technology currently presents the best compromise when it comes to the three metrics; furthermore, the InP HBT technology may be suitable in applications where cost is not the main concern, which is also the case with various other HEMT technologies shown in the figure.

## 5. Other Requirements for Re-Engineering Terahertz Radar Sensors

The third enabling factor for THz research is that THz-capable, modeled active devices must be supported by passive devices, including interconnects and antennas, which are also capable of THz operation.

### 5.1. Passives

Passive devices, specifically inductors and capacitors, tend to exhibit low-quality factors as frequency increases. Therefore, the primary challenge of passive component design in the millimeter-wave, and even more so in the THz regime, is selecting the correct topology and dimension for each component to avoid excessive losses [125]. Numerous alternatives for implementing passive components are possible, including lumped, active and distributed options [38]. In the THz regime, however, only a subset of these options are suitable. Integrated inductors start suffering from excessive losses already well below 100 GHz, while the situation with capacitors is slightly better, but still not satisfactory. Emerging technologies do provide for passive component implementation (e.g., MIM and interdigital capacitors and spiral inductors); however, these are often suitable to be used in the circuit blocks that operate well below *f_T_*. Passives can also be implemented with MEMS modifications, but the frequencies may still be inadequate at present [126]. Hence, the two main directions in THz research are research into transmission-line implementations [127], or alternatively, into implementing passives on different substrates, more suitable for high frequencies [38,128,129,130,131,132].

Transmission lines are suitable for THz integration owing to the small wavelength and the fact that their quality factors increase with frequency. The opposite is, regrettably, true for the insertion loss of the transmission line. Transmission lines can be configured both as inductors and capacitors. Microstrip and CPW transmission lines are two types of printed transmission lines that can be used in nanoelectronic circuits with ease. On discrete substrates, the substrate integrated waveguides (SIW) can also be used [133,134]. A rectangular SIW structure requires two metals and connecting vias to create a waveguide structure. Slow-CPW structures, utilizing various techniques that “slow down” the wave, have been reported in an attempt to increase the transmission line quality factors [135,136]. Nowadays, process design kits for most recent high-speed technologies provide standard transmission line cells for effortless design.

Using substrates geared towards passive component implementation requires the packaging of the designed systems to be reconsidered such that both benefits of integrated substrates and discrete substrates can be used in conjunction with each other, which will be discussed in the next section. Both ceramic and laminate substrates are considered to be sufficiently capable of supporting fabrication of devices operating at least at the millimeter-wave regime. Laminates are fabricated from discrete substrate layers laminated together at high pressure and temperature. Ceramic substrates can be divided into several groups, with low-temperature co-fired ceramics (LTCC) and high-temperature co-fired ceramics (HTCC) suitable for millimeter-wave applications. Other substrates include organics, polymers, including extremely capable liquid-crystal polymers (LCP) with hermetic properties, glasses and hybrid structures.

### 5.2. Interconnects

As wavelengths become ever smaller, interconnects between devices become increasingly difficult to implement. In RF electronics, it is widely considered that a connection longer than about one-tenth of the wavelength starts behaving as a transmission line, and all connections need to be treated as such. Given that technology scaling also results in shorter connections within an IC, this may not be a problem even in the THz regime. Attention, however, needs to be paid to how the transitions between different types of transmission lines are made to minimize the losses [137,138]. Suboptimal device interconnects are known to decrease the apparent values of *f_T_* and *f_max_* by as much as 50% in certain instances [139].

### 5.3. Antennas

The size of the transmitting and receiving antenna, by definition a passive circuit, in a microelectronic system circuit is proportional to the wavelength. Thus, THz antennas are sufficiently small (less than a few millimeters), for antennas and antenna arrays can easily be integrated on chip. Antennas can be placed close to the transmitter or receiver circuitry, allowing them to become an integral part of the IC, without the need to bring the signal from the antenna from outside the package into the package, thus avoiding unnecessary losses [39].

Antenna integration comes with many challenges, however. For example, integrated antennas are prone to coupling to the rest of the RF circuitry, particularly to passives components. On silicon substrates, one of the problems is caused by the fact that a silicon substrate has low resistivity, allowing the signal to find a low-resistivity path through the substrate and degrade the gain of the antenna. Another problem is due to the relatively high dielectric constant of silicon (*ε_r_* = 11.9), which causes substrate absorption [125] and degrades the antenna efficiency. Antennas on other substrates, such as the one deployed in the SOI technology, tend to respond better. At THz frequencies, nevertheless, the benefit of being able to remove the metal interconnects and avoid matching in integrated antennas outweighs many of the above-mentioned drawbacks. Some examples of state-of-the-art integrated antennas can be found in references [140] and [141].

Antennas, like general passive components, can also be implemented on package. Antennas on LTCC, HTCC or LCP (LCP has *ε_r_* = 3.1), for example, can have much higher gains than fully integrated antennas. Large packages also allow for multiple antennas to be readily deployed [142]. Packaged solutions furthermore allow pure MEMS technologies to be utilized, as in the case of the THz helix antenna reported in [14].

## 6. Packaging of Terahertz-Wave Systems

The fourth enabling factor for any research into the THz regime beyond mere proof-of-concept experimentation is the emergence of adequate packaging methodologies. Packaging is necessary to protect circuits from the influences of the environment in which they are deployed.

**Figure 10:**
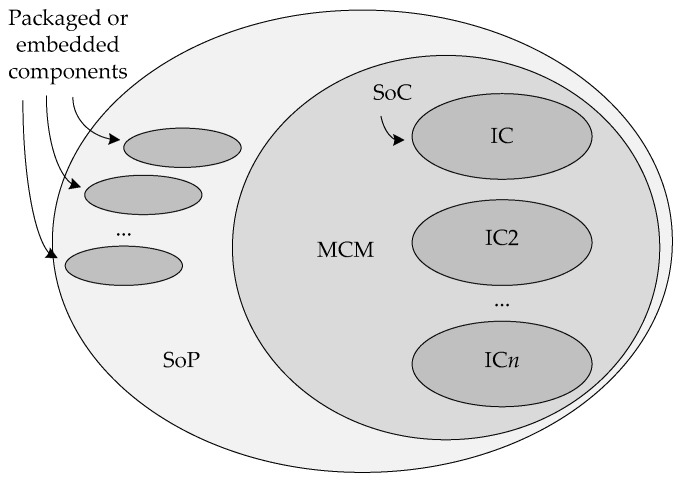
Different packaging options of microelectronic circuits.

Throughout this article, it was generally assumed that re-engineered active THz sensors must be implemented in such a way that the complete system is integrated in a single THz-capable technology, enclosed in an appropriate package. Such an approach is known as system-on-chip (SoC) in literature [38]. It was hinted in the previous section, though, that deployment of other substrates for a more optimal implementation of passive components or antennas, or MEMS-only technologies, can offer a multitude of benefits. In this case, multiple substrates can coexist inside the same package enclosure, with different parts of the system implemented on each, leading to the implementation termed system-on-package (SoP) [128]. If the discrete substrate solely serves as carrier of multiple chips (e.g., any combination SiGe BiCMOS technology, in InP technology and GaAs Schottky diode technology, among others [143], could be an approach to advanced THz systems), then a multi-chip module (MCM) is built. The relationship between SoC, MCM and SoP is illustrated in Figure 10.

For frequencies above 100 GHz, the total cost of a microelectronic system is mostly determined by the packaging and assembly process [144]. Each packaging option comes with several other advantages and disadvantages that must be weighed against one another for a specific system to come to a decision on packaging. Some advantages and disadvantages of SoC and SoP packaging approaches are listed in Table 2. For THz applications, two main considerations are the quality and practicality issues of passive components, and the way in which connections are made between different dies and substrates. If a connection to a high-quality embedded inductor or capacitor can be made without significant losses, then SoP is probably the better option. If satisfactory system performance can be found on a single chip, the lower cost and greater reliability of the single chip solution will be the answer.

The way in which physical interconnects are made in SoC, MCM and SoP is perhaps the most important aspect of THz packaging. In SoC, connections are made from the chip enclosure to the chip substrate, and in MCMs and SoPs, the chip-to-chip interconnects in vertical and horizontal directions, with or without interposers, are also required. Typically, a mix of bonding, bumping and regular or RF TSVs is required. In SoP, most horizontal connections must be made with transmission lines or waveguides, and once again the proper transitions between transmission lines capable of traversing horizontal and vertical directions must be made to minimize losses. In all cases, traditional wire bonding can exclusively be used for direct-current and low-frequency signals; on the contrary, the flip-chip approach deploying solder microbumps, which allows the signals to traverse much shorter paths, is necessary for all RF signals. A 3D coaxial interconnect system, such as the one presented in [145], or electromagnetic transitions, such as the one described for InP HEMTs in [26], may also be required. MEMS circuits can be connected to the supporting substrates using TSVs and small solder bumps and hermetically sealed [146].

Other aspects that need to be considered in conjunction with packaging are heating, heating effects (such as differing thermal expansion of different materials) and associated cooling approaches (especially in high-power systems), electromigration and thermomigration (which become prominent as features decrease), reliability, as well as coupling and shielding [2,38,147]. Modeling and simulation of packaged systems are also much more challenging than those of pure integrated systems, and require deployment of different techniques, most notably ones based on finite-element methods [148].

## 7. Legislative and Commercial Factors

The fifth and last enabling factor for re-engineering THz sensors is legislative and economic factors. As technologies capable of THz applications emerge, first commercial products are typically costly and, in many instances, it does not make sense to push for widespread deployment until the return on investment is deemed satisfactory. In some countries, there could also be legislation prohibiting the deployment of systems at particular frequencies, which could limit the initial rate of acceptance as well.

It is, however, the belief of the authors of this article that these factors can be overcome if other, technical enabling factors are taken care of, and that the benefits of THz technology outweigh the possible hindrances associated with economics and legislation. This is especially true in the case of affordable SiGe BiCMOS technology.

## 8. Review of Reported Terahertz and Low-THz Sub-Systems with the Potential to Be Used with Radar Sensors

In this section, a short review of low-THz and THz subsystems that have been demonstrated at these frequencies is given. These proof-of-concept circuits could potentially serve as a starting point towards the development of re-engineered THz radar systems.

**Figure 11:**
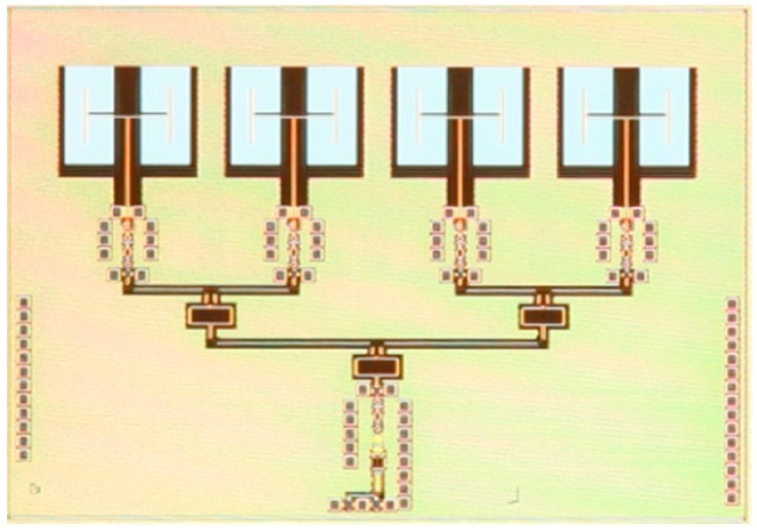
A 245 GHz transmitter array (reproduced with permission from [48]).

Scheytt et al. [46], for example, presented a low-cost radar transceiver operating at 245 GHz, fabricated in IHP (DOTFIVE) technology with *f_max_* = 0.5 THz. The transmitter consists of a 61 GHz VCO with a 122 GHz push-push output, a power amplifier and a frequency doubler to reach a power level of 1 dBm at 245 GHz. On the receiver side, the input signal is passed through a 245 GHz-capable LNA and down-converted with the aid of a VCO tuned to 60 GHz. Even though up-conversion and down-conversion are the main contributions of this paper and the output power is relatively low, the designed four-stage common-base LNA demonstrates the ability of amplification nearing the 300-GHz mark. The transmitter was improved, however, in the paper by Schmaltz et al. [47], showing 7 dBm of power delivered at the same frequency. The transmitter is actually a transmitter array for spectroscopy applications, comprising four branches, each consisting of a power amplifier, frequency doubler and an antenna [48], as illustrated in the microphotograph shown in Figure 11. The same group also demonstrated a spectroscopy transmitter system at 500 GHz (close to *f_max_* of the transistor); however, the output power reached was only −7 dBm [47].

Deng et al. [1] demonstrated an integrated 1 × 4 phased array transmitter intended to operate at 320 GHz in a 0.18 μm SiGe BiCMOS technology. The signal is generated using a VCO at 20 GHz, which is quadrupled to reach 80 GHz. A Wilkinson power divider is then used to split the signal into four branches, and the signal in each branch is passed through two push-push frequency doublers to reach the final frequency of 320 GHz. The power achieved is 10.6 dB.

Reported SiGe transmitters use frequency multiplication to reach (sub-) THz frequencies. Oscillators in such circuits are deliberately designed to have increased power at subharmonics (typically, at double the nominal frequency). The *f_max_* of commercially available InP HBT technologies is higher than that of commercially available BiCMOS technologies, so it comes as no surprise that it is possible to design fundamental oscillators at frequencies of several hundred gigahertz in this technology. Seo et al. [49] presented three varactor-tuned oscillators operating at 310.2, 412.9 and 573.1 GHz in a 0.25 μm InP HBT technology node.

**Figure 12:**
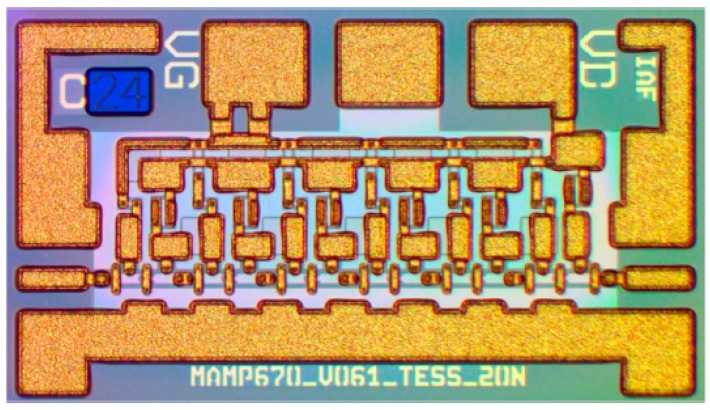
A 600 GHz low-noise application (LNA) in gallium-arsenide (GaAs) metamorphic HEMT (mHEMT) technology (reproduced with permission from [42]).

Several demonstrations of systems in GaAs mHEMT technologies also showed quite attractive results, of which two will be mentioned here. Abbasi et al. [50] demonstrated a 220-GHz active receiver and transmitter for 220-GHz imaging and communication applications, also deploying an integrated antenna. The transmitter and the receiver functions of the chip are performed with the same set of circuits, but the direction that the signal traverses is reversed. Fabrication was completed on GaAs wafers with the metamorphic buffer with composition In*_x_*Al_0.48_Ga_0.52−*x*_, where *x* is an index that transitions from zero to 0.52, as this technology was deemed to be suitable for ultra-low-noise applications. More recently, a six-stage LNA with 150 GHz of bandwidth from 500 to 750 GHz, maximum gain of 15.4 dB and minimum measured noise figure of 15 dB was reported by Tessmann et al. [42]. The wafers had the same composition as in the previous example, as well as T-shaped 20 nm Pt-Ti-Pt-Au transistor gates encapsulated with BCB. Furthermore, the process needed to be modified to increase the quality of CPW lines, including the thinning of the substrate to only 20 μm. Capacitors based on via holes were utilized. The chip photograph of the LNA is shown in Figure 12. The figure shows that the LNA is a six-stage common-source amplifier, complete with power lines and pads. The quality improvement of CPWs results in space saving, and as a result, the whole die consumes an area of only 0.28 × 0.50 mm^2^.

**Figure 13:**
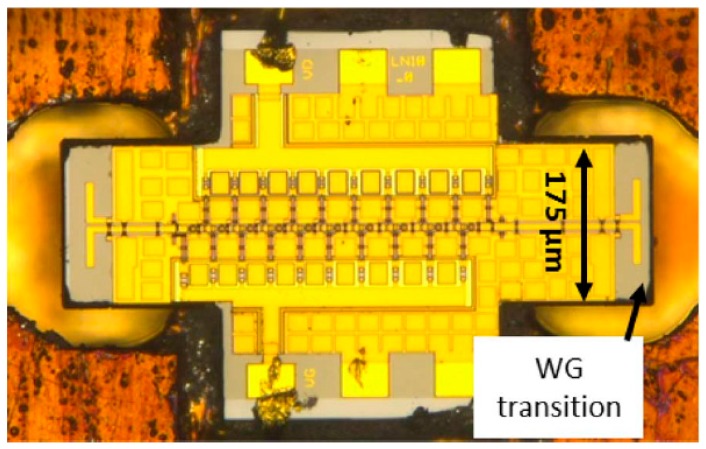
A 850 GHz LNA in InP HEMT technology (reproduced with permission from [52]).

Finally, as expected, amplification at by far the highest frequency was achieved with InP HEMT technology. Mei et al. [51] demonstrated amplification at 1.03 THz, as has already been mentioned. The amplifier was built in the 0.25-nm technology node and consisted of 10 common-source amplifier stages and delivered 9 dB of measured gain. An LNA in the same technology node was demonstrated by Leong et al. [52], delivering 13.6 dB of gain with a noise figure as low as 11.1 dB at 850 GHz. The LNA consisted of 10 amplification stages. Furthermore, to minimize transition losses, custom waveguide transitions (WG transitions) with insertion loss of 1.8 dB were used to connect the input and output of the amplifier to an external waveguide. The complete LNA, including WG transitions and DC pads, is shown in Figure 13.

## 9. Discussion

It is evident that THz-amplification-capable technologies have been available for over a decade, with III-V compound materials leading the way into applications over 1 THz, but with the disadvantage of high manufacturing costs. Less expensive SiGe BiCMOS technologies are considered to be emerging, with transistors incapable of operation above 1 THz, but with a clear roadmap towards 1 THz and above. It is also understood that adequate physics-based simulation models can be extracted for HBT-related technologies, but these usually only become commercially available several years after a technology is reported (which sometimes coincides with the availability of new technology nodes). This review also showed that currently the fastest reported technology, based on InP HEMTs, is not well supported with physics-based models (which is also true for other types of HEMTs), indicating that it will be unrealistic to expect its commercialization in the near future. (However, in addition to huge academic value, exceptions are possible.) With CMOS still barely reaching into the THz range, this leaves SiGe BiCMOS as the main contender for commercial applications of re-engineered radar sensors. Cost is also a major factor in the wide adoption of a technology, and SiGe BiCMOS, fortunately, remains less expensive than the other reviewed technologies [149]. However, the feeling is that with *f_max_* > 0.7 THz, SiGe HBTs still need some frequency-response improvement before it can be said that they can be considered a uniquely capable technology. Improvements are theoretically possible, as indicated in the roadmap as well, supported by the fact that emitter-junction widths can be decreased further from the current size of 110–130 nm and by an introduction of the latest project (a follow-up to DOTSEVEN) aimed at further scaling of SiGe HBTs, named TARANTO [150]. Despite the fact that the silicon-based technology improvements are expected to continue in small increments, many researchers argue that the silicon-based ICs are the best bet to push the field of THz sensing forward [151]. The continuation of this research remains in large part empowered by the cost-effectiveness of silicon-based technologies.

When it comes to passives, interconnects, antennas, and packaging; significant progress has been made in recent years, but it appears, at least when it comes to frequencies of 300 GHz and above, that more research is needed until solutions for these challenges can be standardized. At present, specific design factors drive the choice of passives and packaging, which needs to be supported with intense experimentation, partially because of the complexity of system modeling and simulation. Finally, once all technical challenges have been overcome, commercial factors will also have to be considered.

Nevertheless, with the current approach to THz sensing predominantly relying on bulky and expensive diode mixers or lasers with no amplification capabilities, monolithic active radar sensing solutions, specifically based on silicon-based devices, will soon start appearing. They will, however, have to compete with passive THz detection using solid-state devices configured as detectors operating above *f_max_* values, which will retain superiority above 1 THz.

## 10. Conclusions

In this paper, the emerging transistor technologies capable of amplification in the THz regime were investigated, with the focus on their role in re-engineering THz radar sensors as transceivers. With III-V compound-material technologies leading the way into applications over 1 THz, relatively closely followed by the less expensive SiGe BiCMOS technology and with numerous demonstrated oscillator and amplifier circuits in a range of technologies, it appears that future prospects are good. However, the situation is not so simple. The availability of technologies is not the only prerequisite when it comes to commercialization. Commercialization requires stable empirical models for conceptual circuit designs, as well as simulation models for rapid design at particular technology nodes. Furthermore, technology researchers also tend to focus on developing models for active devices only, with less attention being paid to passive components and packaging, despite them also being determining factors for commercialization of potential products. Finally, there may be other commercial factors hindering the widespread adoption of re-engineered THz sensors, including cost and spectrum allocation and legislative challenges. In this paper, it was argued that as advanced technologies become more readily available, the interest of the academic and subsequently the commercial community in the vast field of THz applications is increasing and that it can be expected that active radar sensing solutions will soon start taking over a fair share of the commercial market.

## Figures and Tables

**Table 1:** Properties of various semiconductors (taken from [60,61,62,63]).

Property	Silicon (Si)	Gallium Nitride (GaN)	Silicon Germanium (SiGe)	Gallium-Arsenide (GaAs)	Indium Phosphide (InP)
Bandgap energy (eV)	1.12	3.42	0.945	1.42	1.34
Electron mobility (cm^2^/V-s)	1360	2000	7700	8500	5400
Breakdown of electric field (V/cm)	2 × 10^5^	3.5 × 10^6^	4 × 10^5^	4 × 10^5^	5 × 10^5^
Saturation electron drift velocity (cm/s)	10^7^	2.5 × 10^7^	13.5	1.2 × 10^7^	2 × 10^7^
Relative dielectric constant (*ε_r_*)	11.7	9	-	12.9	12.5

**Table 2:** Advantages and disadvantages of system-on-chip (SoC) and system-on-package (SoP).

Type of Implementation	Advantages	Disadvantages
SoC	High integration Small size Inexpensiveness	Low-quality passives Thermal issues
SoP	Fairly good integration High density Higher quality passives	Larger than that of the SoC Longer interconnects Longer time to market Higher production costs Modeling simulation, reliability and yield considerations
